# The Dutch Early-Stage Melanoma (D-ESMEL) study: a discovery set and validation cohort to predict the absolute risk of distant metastases in stage I/II cutaneous melanoma

**DOI:** 10.1007/s10654-024-01188-4

**Published:** 2025-01-09

**Authors:** Catherine Zhou, Antien L. Mooyaart, Thamila Kerkour, Marieke W. J. Louwman, Marlies Wakkee, Yunlei Li, Quirinus J. M. Voorham, Annette Bruggink, Tamar E. C. Nijsten, Loes M. Hollestein

**Affiliations:** 1 Department of Dermatology, Erasmus MC Cancer Institute, Rotterdam, The Netherlands; 2 Department of Pathology, Erasmus MC Cancer Institute, Rotterdam, The Netherlands; 3 Department of Research and Development, Netherlands Comprehensive Cancer Organization, Utrecht, The Netherlands; 4 Department of Pathology and Clinical Bioinformatics, Erasmus MC Cancer Institute, Rotterdam, The Netherlands; 5 Dutch Nationwide Pathology Databank (Palga), Houten, The Netherlands

**Keywords:** Melanoma, Risk prediction, Biomarkers, Multi-omics, Population-based, Prognostic

## Abstract

Early-stage cutaneous melanoma patients generally have a favorable prognosis, yet a significant proportion of metastatic melanoma cases arise from this group, highlighting the need for improved risk stratification using novel prognostic biomarkers. The Dutch Early-Stage Melanoma (D-ESMEL) study introduces a robust, population-based methodology to develop an absolute risk prediction model for stage I/II melanoma, incorporating clinical, imaging, and multi-omics data to identify patients at increased risk for distant metastases. Utilizing the Netherlands Cancer Registry and Dutch Nationwide Pathology Databank, we collected primary tumor samples from early-stage melanoma patients, with and without distant metastases during follow-up. Our study design includes a discovery set of metastatic cases and matched controls to identify novel prognostic factors, followed by a validation cohort using a nested case–control design to validate these factors and to build a risk prediction model. Tissue sections underwent Hematoxylin & Eosin (H&E) staining, RNA sequencing (RNAseq), DNA sequencing (DNAseq), immunohistochemistry (IHC), and multiplex immunofluorescence (MxIF).The discovery set included 442 primary melanoma samples (221 case–control sets), with 46% stage I and 54% stage II melanomas. The median time to distant metastasis was 3.4 years, while controls had a median follow-up time of 9.8 years. The validation cohort included 154 cases and 154 controls from a random population-based selection of 5,815 patients. Our approach enabled the collection of a large number of early-stage melanoma samples from population-based databases with extensive follow-up and a sufficient number of metastatic events. This methodology in prognostic cancer research holds the potential to impact clinical decision-making through absolute risk prediction.

**Supplementary Information:**

The online version contains supplementary material available at 10.1007/s10654-024-01188-4.

## Introduction

Cutaneous melanoma stands as a notably aggressive form of skin cancer, resulting in poor outcomes once it metastasizes to distant sites [1]. Although more than 80% of the patients are diagnosed with early-stage, locally invasive disease, which typically forecasts a favorable prognosis, up to 29% still progress to a distant metastatic stage [2, 3]. This highlights an unrecognized propensity for metastases in this subset. Epidemiological studies have further highlighted that 41% of the patients who die of melanoma and 60% of the patients with distant metastatic disease, presented with stage I or II disease initially [4, 5]. This indicates that the current staging system by the American Joint Committee of Cancer (AJCC), which relies on the Breslow thickness and the presence of ulceration, lacks accuracy in identifying these early-stage patients with aggressive course of disease [1]. Given the high and rising incidence of early-stage melanoma, the absolute number of patients who die from the disease is substantial. Of all 57,000 melanoma deaths worldwide each year, an estimated 23,000 are attributed to patients who were initially diagnosed with early-stage melanoma [6–8].

The advancements in systemic therapies, including targeted therapies and immune checkpoint inhibitors, have significantly improved the survival rates for metastatic melanoma [2, 9]. Recent clinical trials indicate that these benefits extend to stage IIB and IIC patients [10–13]. However, the AJCC staging system falls short in guiding adjuvant therapy selection, as patients with stage IIIA disease appear to have a more favorable prognosis than those with stages IIB and IIC [1]. This emphasizes the need for improved risk stratification tools to identify the patients who are at risk for metastases. Effective risk stratification could personalize patient management, directing systemic therapies to the patient likely to benefit from them.

The need for robust prognostic markers for early-stage melanoma is therefore critical. Well-known patient-and tumor characteristics are age at diagnosis, sex, the presence of ulceration in the primary tumor and the Breslow thickness [14–17]. Other pathological predictors, such as mitotic rate and Clark level, lack sufficient strength or reliability [1, 18]. Publicly available nomograms based on clinicopathological factors have been developed to predict sentinel lymph node (SLN) positivity, recurrence-free survival in thin melanomas and in SLN-negative patients [19–22]. These nomograms focus on SLN outcome or require the SLN status and/or mitotic rate, making them less than optimal for patients with very thin melanomas without an indication for a SLN biopsy (SLNB).

The identification of molecular characteristics in the resected primary melanoma could refine prognostic assessments, including genetic mutations, gene expression levels, immunological markers and epigenetic changes [23]. Although there are significant collections of samples, such as those from the Cancer Genome Atlas, the InterMEL Consortium and the BioMEL biobank, and advancements in gene expression profiling have been noted, the effective application of these insights is hampered by the scarcity of metastatic events in early-stage patients [24–30]. This infrequent event, coupled with the wide variance in the timing of distant recurrence (< 1 year to more than 8 years), leads to a lack of comprehensive collections of early-stage melanoma samples from patients who have subsequently developed distant metastases during follow-up [2].

In this study, we introduce an epidemiological study design that facilitates the gathering of a significant collection of tumor samples from patients with an extensive follow-up period and a sufficient number of metastatic events, using population-based clinical and pathological data. This study design enables to include clinical data, imaging data and RNA and DNA sequencing (RNAseq, DNAseq) data. Our study enables the integration of clinical, imaging and multi-omics data to construct an absolute risk prediction model capable of identifying patients at elevated risk for distant metastases. This epidemiological study design serves as a robust and adaptable framework for prognostic research, particularly well-suited for studies focusing in rare outcomes.

## Methods

### Data sources

Our study leverages the nationwide database of the Netherlands Cancer Registry (NCR) with long-term follow-up linked to the Dutch Nationwide Pathology Databank (Palga), and samples collected from the pathology archives via the Dutch National Tissue Portal (DNTP). Cutaneous melanoma in the Netherlands is routinely registered by the Netherlands Cancer Registry (NCR). This registry is embedded in the Netherlands Comprehensive Cancer Organization. The NCR registers newly diagnosed malignancies upon automated notifications by Palga since 1989 [31].

For this study, we used data on patients with cutaneous melanoma who were diagnosed with stage I or II disease at initial diagnosis. The retrieval was based on morphology codes M8720-8790 and topography code C44 of the third edition of the International Classification of Diseases for Oncology (ICD-O3) [32]. Melanomas of unknown primary site (C80.9) and patients with multiple melanomas were excluded. Trained data managers collected the data from pathology reports and digital patient records. We retrieved the following variables from the NCR: sex, age at diagnosis, year of diagnosis, vital status, topography, morphology, Breslow thickness, ulceration, clinical and pathological tumor-node-metastasis (TNM) stage, AJCC stage and SLNB. The AJCC stage used corresponded to the version valid at the time of diagnosis: AJCC 6 for 2009 and earlier, AJCC 7 for 2010–2017, and AJCC 8 from 2018 onward. Since July 2017, the NCR registers disease progression to stage III and IV during follow-up. For melanomas diagnosed before this date, we used data from Palga to obtain information about disease progression, including incidence dates and localizations of loco-regional (stage III) and distant recurrences (stage IV). Follow-up of recurrence and vital status of all patients in the study was updated until February 2024.

The registration of disease progression includes both patients with histopathologically confirmed and non-histopathologically confirmed metastases (e.g. observed with imaging, based on digital patient records) since 2017. Prior to this period, data were limited to cases with histopathologically confirmed metastases, potentially introducing selection bias. To address this, we expanded our patient selection to include individuals who, after initially being diagnosed with stage I or II disease, were later found to have distant metastases as per the digital patient records from the Erasmus MC Cancer Institute prior to 2017. Data and material were retrieved using linkages with aforementioned databases.

The Dutch Nationwide Pathology Databank (Palga) facilitates the use of nationwide histo- and cytopathology data by its decentralized information system, central databank and dedicated communication and information exchange infrastructure [33]. The registry was set up in 1971, under the name Palga (“*Pathologisch Anatomisch Landelijk Geautomatiseerd Archief*”). Nowadays, it encompasses all pathology laboratories in the Netherlands. Excerpts of the pathology reports are transferred to the central databank. Encrypted patient identifiers and demographic data are included. Based on the excerpts, a structured and coded Palga diagnosis is formed based on topography, morphology, function, procedure and disease.

The Dutch National Tissue Portal (DNTP) was used to request the selected tumor material. The DNTP utilizes Palga’s nationwide network and facilitates the use of tissue samples for research [34]. The DNTP consists of a centrally organized internet portal to request (tumor)tissue material. Dedicated employees provide practical support at peripheral pathology departments in the Netherlands.

The use of left-over diagnostic tissue samples for scientific research is based on the ‘no objection’ principle, as stated in the Code of Conduct for Health Research from the Committee on Regulation of Health Research. Consequently, a waiver of informed consent was granted [35].

### Study designs

We established a discovery set using a matched case–control design, and a validation cohort using a nested case–control design. In the discovery set, early-stage melanoma patients who developed distant metastasis during follow-up, were matched based on staging and well-known prognostic variables. We chose distant metastasis as the main outcome because these patients are at a higher risk of disease-specific death and represent the target group for systemic therapy among stage I/II melanoma patients. The aim of the matching approach is to identify novel prognostic factors that are independent of the already well-established prognostic factors.

**Fig. 1:**
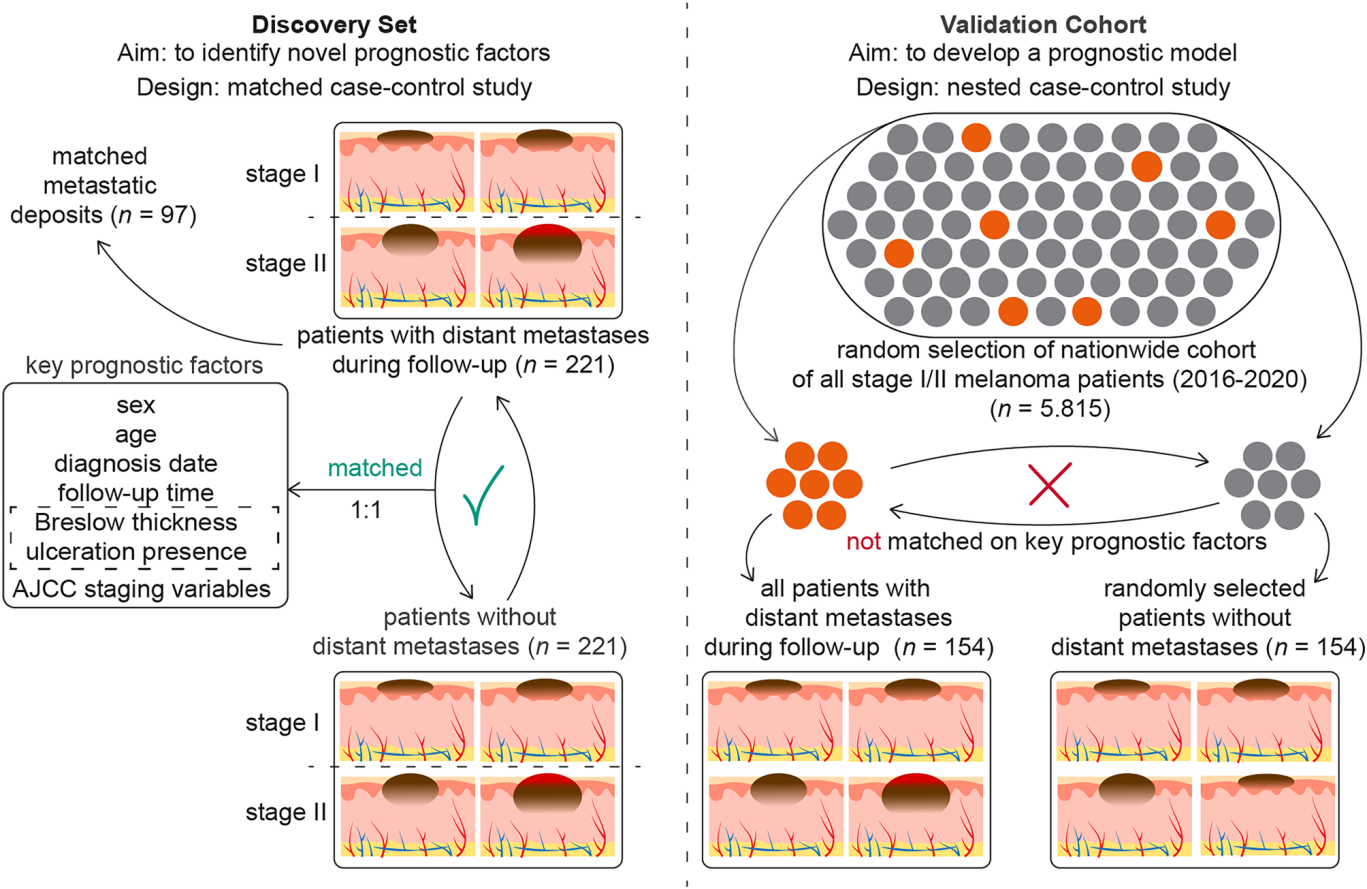
Matched case–control design of the discovery set and nested case–control design of the validation cohort. (Abbreviations: AJCC = American Joint Committee of Cancer)

To validate these novel factors and to build a prognostic model, we created a validation cohort structured as a nested case–control study derived from a comprehensive nationwide cohort. This validation cohort will be used to develop a clinical prediction model that incorporates both known and newly identified prognostic factors from the discovery set. If the final sample size of the validation cohort is sufficient, it may be divided into two parts: one for the development of the clinical prediction model and the other for its external validation. The nested case–control design enables the calculation of absolute risk probabilities associated with these novel factors, providing a measure more relevant than relative risk for clinical practice. Such a framework enhances the practicality and effectiveness of our prediction model in real-world clinical scenarios [36, 37]. A schematic overview of the study designs is provided in Fig. 1.

### Discovery set

A matched case–control study was conducted involving adult patients diagnosed with a single stage I or II melanoma. Patients who developed distant metastases during follow-up (i.e. cases) were matched with those who did not (i.e. controls). Matching criteria included the AJCC 8 staging variables—Breslow thickness and presence of ulceration—along with age at diagnosis and sex, due to their established prognostic significance [14–17].

To identify cases, we initially selected all adult patients who progressed from stage I and stage II disease to distant metastasis between 2013 and 2019 based on digital patient records from the Erasmus MC Cancer Institute. Given the relative infrequency of distant metastases following stage IA and IB diagnoses, we expanded our selection to include patients from the NCR, to supplement those from the Erasmus MC Cancer Institute. No restrictions were placed on the time to metastases for cases, thereby including both tumors that metastasized early and those that progressed later (e.g., 5–10 years post primary diagnosis). We aimed for an equal distribution of cases across stages I and II to facilitate stratified analyses. To ensure the most complete follow-up data, we requested updates on locoregional and distant disease progression from the NCR, along with the vital status of patients, in February 2024.

Matching on categorical variables was exact. For continuous variables, matching was categorized and prioritized as follows: Breslow thickness, age, and follow-up time. Specifically, a case diagnosed with distant metastases after *x* days was matched to a control whose follow-up time was equal to or greater than *x* days. Breslow thickness matching used intervals of 0.25 mm for up to 2.0 mm, 0.50 mm for 2.00–4.00 mm, and 1.00 mm for 4.00 to 10.00 mm, with no specific intervals for thicknesses greater than 10.00 mm. Age was matched within 5-year bands. An example of prioritizing matching variables could be illustrated by a 62-year-old case with a Breslow thickness of 1.4 mm being matched to a 67-year-old control who also has a Breslow thickness of 1.4 mm, instead of a 63-year-old control with a Breslow thickness of 1.3 mm.

After selecting cases and their matched controls, we linked the data from the NCR to Palga to obtain complete pathological histories for the selected patients. This included data on the primary melanoma and any histopathologically confirmed loco-regional or distant metastases. We manually reviewed pathology reports and excluded any patients with multiple melanomas, non-cutaneous melanomas, those initially diagnosed at stage III, or those with unclear pathology reports concerning their primary melanoma. Despite initially filtering for stage I and II patients with a single primary melanoma within the NCR, a few cases with multiple melanomas were included in the selection and subsequently excluded after reviewing the pathology data. Initially, cases were matched to controls at a 1:10 ratio to ensure that at least one suitable control could be selected from the pathology records and to provide flexibility in selecting a new control if the formalin-fixed paraffin-embedded (FFPE) tumor sample could not be retrieved. We ultimately selected one control per case for the retrieval of tumor samples and further analysis.

### Validation cohort

The validation cohort comprised adult patients diagnosed with a single stage I or II melanoma between 2016 and 2020 in the NCR. We randomly selected a sample from this source cohort large enough to ensure a minimum of 400 eligible cases. These selected patients were then linked to Palga to retrieve complete pathological histories. From this pool, we identified cases and matched them to controls at a 1:5 ratio, ensuring the retrieval of FFPE tumor material for one control per case. Similar to the discovery set, pathology reports for all cases and potential controls were reviewed and selected. Based on the proportion of approved cases of the eligible ~ 400 cases, we calculated the size of the nationwide source cohort, which is a random sample from the nationwide stage I/II melanoma cohort.

Unlike the discovery set, we did not match the cases and on controls on age, sex, Breslow thickness and presence of ulceration, but did for follow-up time, as required for a nested case–control design to calculate absolute risks [36]. Additionally, based on differentially expressed genes identified during the quality control phase of the discovery set, we matched cases and controls on the type of surgical procedure (punch or shave biopsy vs. elliptical excision). For logistic efficiency, we also matched cases and controls based on the pathology lab of origin. This approach was not intended to address potential biases, as no such biases related to the pathology lab were observed in the RNA sequencing data from the discovery set. Each case was matched with one control for further processing. The retrieval and sectioning process of tumor specimens for the validation cohort mirrored that of the discovery set.

### Tumor sample processing

We obtained FFPE tumor specimens, corresponding Hematoxylin & Eosin (H&E) slides, and anonymized pathology reports of the primary melanoma from all contributing pathology laboratories via the DNTP. To avoid the effects of tissue alterations from previous biopsies, we used tumor specimens from the initial surgical procedure of the primary melanomas.

From the H&E slides provided by the originating pathology laboratories, one FFPE block that best represented the tumor for further processing was selected. Each chosen block was then sectioned to produce a new H&E slide, which was subsequently digitalized. A dermatopathologist (A.M.) reviewed these digitalized slides to confirm the representativeness of the melanoma and to ensure the accuracy of the recorded Breslow thickness and ulceration status. If discrepancies were noted between our H&E slides and the original data, we reassessed the H&E slides from the original laboratory. If inconsistencies persisted and it was not feasible to select an alternative control, we excluded the set from further analysis.

**Fig. 2:**
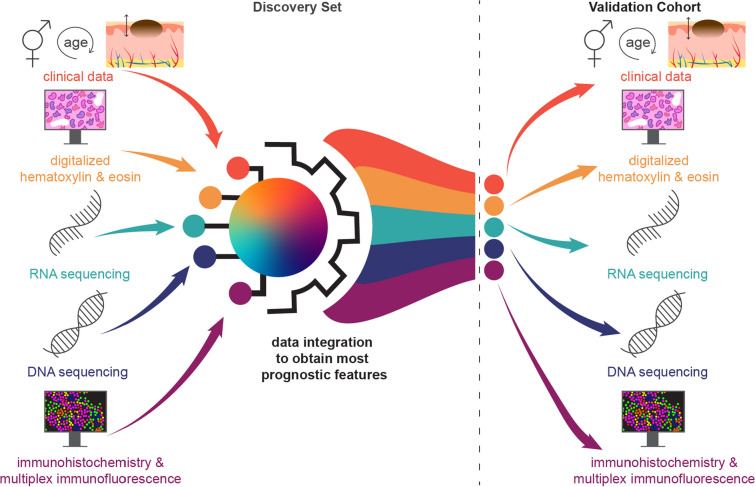
Clinical, imaging, RNA sequencing and DNA sequencing data derived from the discovery set will be integrated and the most prognostic features will be validated in the validation cohort

To develop a prognostic model integrating multi-omics, imaging, and clinical data, we prepared slides from each sample for H&E staining, RNAseq, DNAseq, immunohistochemistry (IHC), and multiplex immunofluorescence (MxIF) from both the discovery set and the validation cohort (Fig. 2).

We sectioned 4 µm slides from the selected FFPE blocks, alternating the sectioning order to ensure uniform representation of the entire tumor sample (Supplementary Information 1). To prevent contamination, a new blade was used for each section. Slides designated for IHC and MxIF were dipped in 60° Celsius paraffin to preserve tissue integrity. RNA was extracted from whole slides to include RNA from both tumor cells and surrounding immune cells, enhancing our analysis of the tumor microenvironment. RNA isolation was conducted as promptly as possible, within one week for most samples, to prevent degradation. For DNAseq, we performed macrodissection to predominantly obtain DNA from tumor cells. Given DNA’s greater stability, no strict timing requirements were applied before DNA isolation. Details regarding the RNA and DNA isolation and sequencing processes will be provided in separate manuscripts, as they fall outside the scope of this epidemiological design description.

In the discovery set, each FFPE block yielded 54 slides: 11 for H&E staining, 10 for DNAseq, 13 for RNAseq, 3 for IHC and 17 for MxIF. The first slide was always an H&E slide, which was digitalized to assess initial tumor representativeness and to record various histopathological variables. For each set of 10 MxIF slides, an adjacent H&E slide provided a morphological reference, facilitating more precise analyses. Similarly, from the validation cohort, we processed 53 slides per FFPE block: 10 for H&E, 10 for DNAseq, 13 for RNAseq, and 20 for IHC or MxIF, with the first 10 MxIF/IHC slides accompanied by an adjacent H&E slide (Supplementary Information 1).

To explore the clonal heterogeneity between primary melanomas and their corresponding distant metastatic deposits, we identified all histopathologically and cytopathologically confirmed distant metastases, as recorded by Palga, utilizing the detailed pathological histories available to us from the discovery set. Following the procedure established for primary tumors, we procured FFPE blocks and cytological slides from the DNTP. For each sample, the H&E slide was reviewed by a pathologist (A.M.) to confirm the presence of at least 20% tumor area, and 10 slides were generated for DNA isolation. Investigating the genetic relationships between primary tumors and their metastases will help in identifying and excluding cases where the distant metastases may not be directly derived from the identified primary melanoma, suggesting the presence of another, unknown primary tumor.

### Statistical analyses

The sample size for our discovery set was derived from the requirements of developing an RNA gene expression profile. Lacking specific features and effect sizes, we referenced a previous study that successfully developed a gene expression profile to predict low risk of SLN metastasis among melanoma patients [38]. In this previous study, differential gene expression analyses were conducted on 6 samples, which led to identifying 54 candidate genes tested across 160 patients. To enhance our capability to study all expressed genes comprehensively, we doubled the initial sample size from the referenced study, aiming for at least 350 patients, with 175 patients who developed distant metastases and 175 who did not. This sample size ensures at least 80% power to detect prognostic factors with an odds ratio (OR) of ≥ 2 occurring at a frequency of 20%, or an OR of ≥ 3 for factors occurring at a frequency of 5%.

The validation cohort comprises a sufficient number of events for robust model development, as this determines the effective sample size. Although various thresholds exist for the number of events per variable in different contexts, at least 10 events per variable are generally recommended for accurate prediction modeling of binary outcomes [39, 40]. Achieving this ratio requires shrinkage of the regression coefficients in the final model to prevent overfitting. Therefore, we aimed to include at least 100 cases and 100 controls in the nested case–control design of the validation cohort. With 10 events per variable, we can allocate 10 degrees of freedom in a regression model, which is adequate to incorporate known predictors (age, sex, Breslow thickness, and ulceration) along with novel prognostic factors identified from a pre-trained model of the discovery set.

We intentionally oversampled the initial validation cohort and requested a higher number of FFPE tumor specimens to ensure that a sufficient sample size was achieved within project timelines. Upon receiving and evaluating the available cases, we adjusted the size of the nationwide source cohort proportionally based on the percentage of cases received—for instance, if only 50% of the expected cases were obtained, we then randomly sampled 50% from the baseline cohort.

The descriptive statistics of the distribution of the clinical characteristics for both the discovery set, the validation cohort and the nationwide source cohort are presented. Differences in age, sex, Breslow thickness, presence of ulceration, AJCC stage, body site of the primary melanoma, morphological subtype and performed SLNB’s between cases and controls were tested. Categorical variables were analyzed using the McNemar’s test or the McNemar-Bowker test for > 2 categories, and continuous variables were assessed using the Wilcoxon signed-rank test, all accounting for the paired nature of the data. The level of statistical significance was set at a two-sided* p* < 0.05.

Statistical analyses were conducted using SAS® (9.4 M8), R Studio (version 4.3.3), and IBM® SPSS® software (version 29.0).

## Results

### Sample collection

**Fig. 3:**
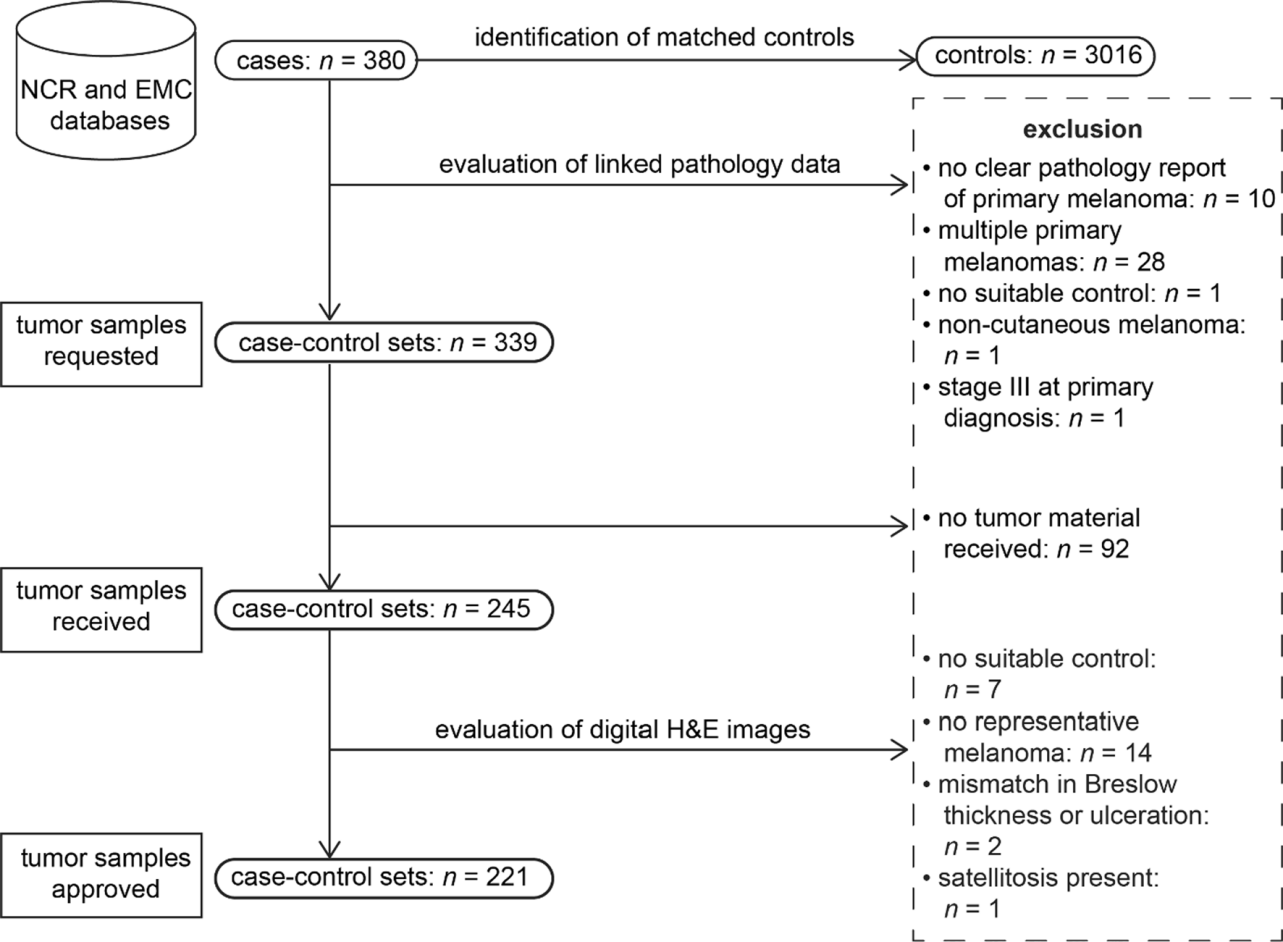
Selection process of the matched case–control sets in the discovery set. (Abbreviations: NCR = Netherlands Cancer Registry, EMC = Erasmus MC Cancer Institute, H&E = Hematoxylin & Eosin)

The selection process for the discovery set began by identifying 380 eligible cases from the NCR and Erasmus MC Cancer Institute databases (Fig. 3). Following the review of pathology reports, FFPE tumor samples were requested for 339 case–control sets, from which samples for 245 sets were received. Subsequent evaluation of the digital H&E images by a dermatopathologist (A.M.) led to the approval of 221 case–control sets for further analysis.

**Fig. 4:**
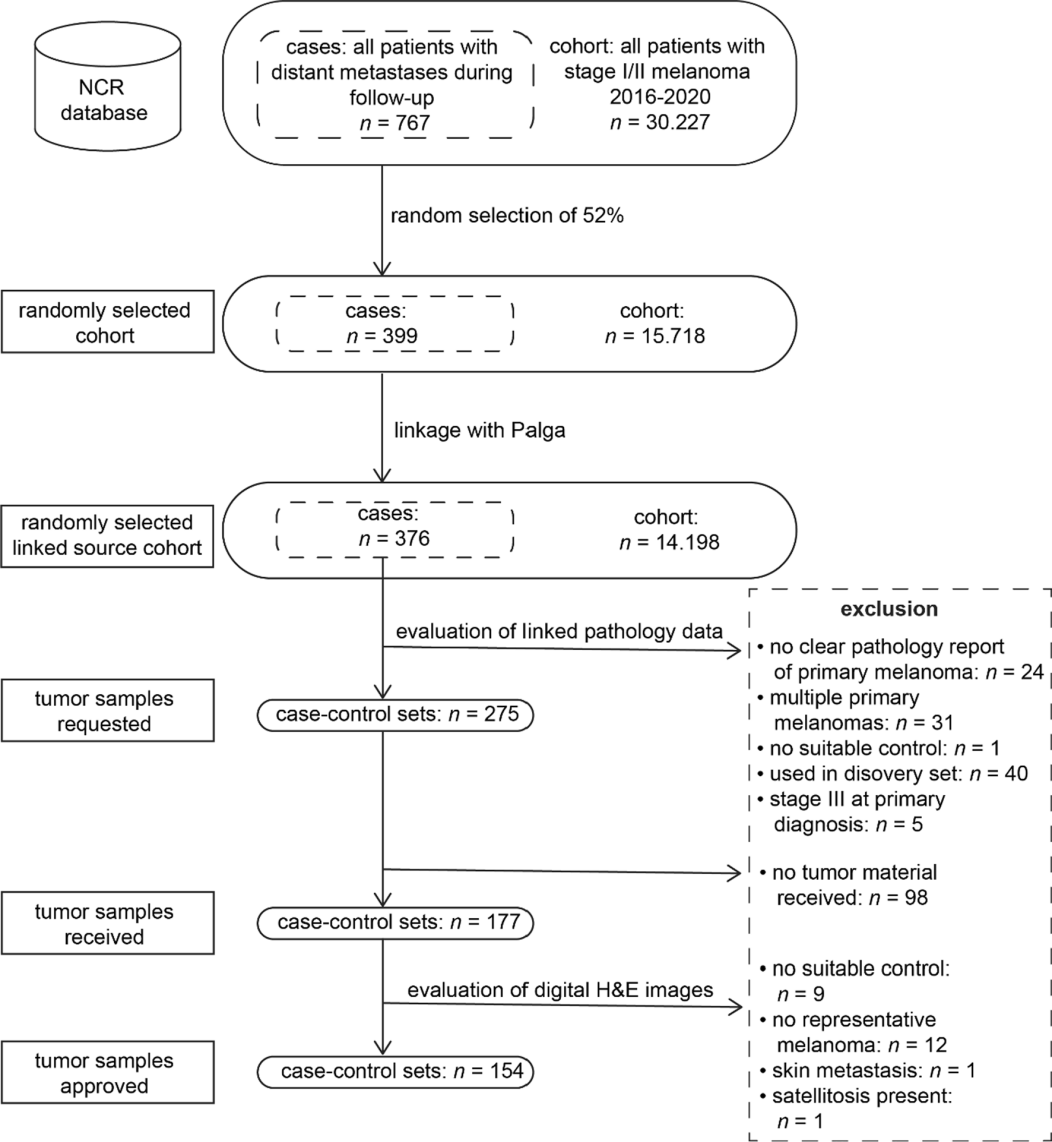
Selection process of the nested case–control design of the validation cohort (Abbreviations: NCR = Netherlands Cancer Registry, Palga = Dutch Nationwide Pathology Databank, H&E = Hematoxylin & Eosin)

For the nested case–control design of our validation cohort, we identified all patients with stage I/II melanoma between 2016 and 2020 registered in the NCR, totaling 30,227 individuals (Fig. 4). Within this nationwide cohort, 767 patients (2.5%) progressed to distant metastases during follow-up. A calculated random selection of 52% resulted in a cohort of 15,718 patients, which included 399 patients with distant metastases. After data linkage with Palga, a cohort of 14,198 remained, with 376 qualifying as cases for our study. Pathology data of all the 376 cases was evaluated, and 275 eligible cases were matched to controls. Tumor samples were requested for these 275 case–control sets, with 177 sets received and 154 sets approved based on the H&E images for inclusion in the study. The main reason was the absence of a representative invasive melanoma on the H&E image, likely due to the tissue in the blocks being depleted for diagnostic purposes. The final validation cohort thus comprised 154 case–control sets, derived from a calculated random pool of 5,815 individuals with stage I/II melanoma.

### Clinical characteristics

Our discovery set consisted of 221 cases and an equal number of matched controls of which 61% (*n* = 268) were male with a median age of 64 years (IQR: 50–71). Breslow thickness was nearly identical with a median of 2.0 mm (IQR: 1.0–3.4 mm, min–max range: 0.4–15.5) for cases and 1.9 mm (IQR: 1.1–3.3 mm, min–max range: 0.3–20.0) for controls and 33% (*n* = 146) of the tumors were ulcerated. Twenty-four percent of the tumors had a Breslow thickness of ≤ 1.0 mm. The distribution between stages I (46%, *n* = 101) and II (54%, *n* = 120) was fairly balanced (Table 1).

**Table 1:** Clinical characteristics of the discovery set, the validation cohort and its nationwide source cohort

	Discovery set			Validation cohort			
				Nationwide source cohort	Random selection from source cohort		
Characteristic	Cases (*n* = 221)	Controls (*n* = 221)	*p-*value	Total (*n* = 5,815)	All cases from source cohort (*n* = 154)	Randomly selected controls (*n* = 154)	*p*-value
Sex (*n* (%))							
Male	134 (61)	134 (61)	> 0.99	2,837 (49)	83 (54)	65 (42)	0.05
Female	87 (39)	87 (39)		2,978 (51)	71 (46)	89 (58)	
Age, years (median (IQR))	64 (50–71)	63 (52–71)	0.43	62 (51–73)	68 (55–77)	63 (53–72)	0.01
Breslow thickness, mm (median (IQR, min–max range))	2.0 (1.0–3.4, 0.4–15.5)	1.9 (1.1–3.3, 0.3–20.0)	0.70	0.8 (0.5–1.4, 0.1–60.0)	2.7 (1.3–4.6, 0.3–32.0)	0.8 (0.5–1.3, 0.2–14.5)	< 0.001
Breslow thickness, mm (*n* (%))							
< 1.0	54 (24)	54 (24)	> 0.99	3,744 (64)	25 (16)	95 (62)	< 0.001
> 1.0–2.0	58 (26)	59 (27)		1,120 (19)	37 (24)	38 (25)	
> 2.0–4.0	75 (34)	73 (33)		559 (10)	40 (26)	14 (9)	
> 4.0	34 (15)	35 (16)		384 (7)	52 (34)	7 (5)	
Ulcerated (*n* (%))	73 (33)	73 (33)	> 0.99	533 (9)	68 (44)	12 (8)	< 0.001
Stage (*n* (%))							
IA	40 (18)	40 (18)	> 0.99	3,595 (62)	20 (13)	89 (58)	< 0.001
IB	61 (28)	61 (28)		1,209 (21)	36 (23)	41 (27)	
IIA	44 (20)	44 (20)		487 (8)	28 (18)	13 (8)	
IIB	55 (25)	55 (25)		336 (6)	32 (21)	10 (6)	
IIC	21 (10)	21 (10)		188 (3)	38 (25)	1 (1)	
Body site (*n* (%))							
Face/neck	15 (7)	12 (5)	0.06	453 (8)	14 (9)	6 (4)	0.48
Scalp	21 (10)	14 (6)		321 (6)	14 (9)	7 (5)	
Upper extremities	38 (17)	48 (22)		1,333 (23)	28 (18)	34 (22)	
Trunk	108 (49)	98 (44)		2,303 (40)	59 (38)	64 (42)	
Lower extremities	39 (18)	49 (22)		1,400 (24)	39 (25)	43 (28)	
Morphological subtype (*n* (%))							
Superficial spreading	141 (64)	147 (67)	n/a	4,634 (80)	100 (64)	132 (86)	n/a
Nodular	56 (25)	58 (26)		452 (8)	41 (27)	13 (8)	
Acral lentiginous	1 (0)	1 (0)		55 (1)	4 (3)	0 (0)	
Lentigo maligna melanoma	4 (2)	1 (0)		312 (5)	4 (3)	2 (1)	
Amelanotic melanoma	1 (0)	0 (0)		7 (0)	0 (0)	2 (1)	
Desmoplastic melanoma	1 (0)	0 (0)		35 (0)	0 (0)	1 (1)	
Unspecified	17 (8)	14 (6)		320 (6)	5 (3)	4 (3)	
Surgical procedure (*n* (%))							
Elliptical excision	198 (90)	210 (95)		n/a	137 (89)	137 (89)	
Shave or punch biopsy	23 (10)	11 (5)		n/a	17 (11)	17 (11)	
Sentinel lymph node biopsy (*n*(%))			0.03				0.01
Performed, overall	70 (32)	93 (42)		1893 (33)	72 (47)	49 (32)	
Not indicated, i.e. stage pT1a	32 (15)	33 (15)		2842 (49)	11 (7)	66 (43)	
Eligible but not performed (stage pT1b or higher)	120 (64)	96 (52)		1102 (37)	71 (50)	41 (47)	
Time until stage IV recurrence, years (median (IQR, min–max range))	3.4 (1.7–5.4, 0.2–11.0)	n/a		1.8 (1.0–3.3, 0.3–5.3)	1.8 (1.0–3.3, 0.3–5.3)	n/a	
Time until stage IV recurrence (n(%))							
< 2 years	71 (32)	n/a		85 (55)	85 (55)	n/a	
2–5 years	83 (38)	n/a		66 (43)	66 (43)	n/a	
5–8 years	51 (23)	n/a		3 (2)	3 (2)	n/a	
> 8 years	16 (7)	n/a		0 (0)	0 (0)	n/a	
Alive survival status at last follow-up (*n* (%))	58 (26)	173 (78)		5029 (87)	44 (29)	143 (93)	
Follow-up duration until censoring or death, years (median (IQR))	5.9 (3.4–8.8)	9.8 (7.4–12.3)		5.3 (4.0–6.6)	3.5 (1.9–5.4)	6.1 (4.8–7.3)	
Follow-up duration until censoring or death, years (*n* (%))							
< 2 years	28 (13)	6 (3)		297 (5)	42 (27)	3 (2)	
2–5 years	62 (28)	12 (5)		2348 (40)	66 (43)	39 (25)	
5–8 years	68 (31)	48 (22)		3115 (54)	46 (30)	111 (72)	
> 8 years	62 (28)	154 (70)		52 (1)	0 (0)	1 (1)	
Disease progression after initial follow-up period (*n* (%))							
Stage III	n/a	24 (11)		n/a	n/a	5 (3)	
Stage IV	n/a	20 (9)		n/a	n/a	1 (1)	

Most melanomas were on the trunk and frequently manifesting as superficial spreading melanoma, followed by nodular subtype. SLNB was performed in 32% of the cases (*n* = 70) and 42% of the controls (*n* = 93). Despite being eligible base on the guidelines (i.e. stage pT1b or higher), 64% of the cases (*n* = 120) and 52% of the controls (*n* = 96) did not undergo an SLNB (Table 1).

The median time to progression to stage IV was 3.4 years (IQR: 1.7–5.4 years, min–max range: 0.2–11.0), with 70% (n = 154) of cases experiencing distant recurrence within 5 years, and 30% having a longer progression time. At the last follow-up, 26% (*n* = 58) of the cases were alive, compared to 78% (*n* = 173) of the controls. The median follow-up time until censoring due to death was 5.9 years (IQR: 3.4–8.8 years) for cases and 9.8 years (IQR: 7.4–12.3 years) for controls, with 92% of the controls (*n* = 202) having at least 5 years of follow-up data. Eleven percent (*n* = 24) of the controls progressed to stage III and 9% (*n* = 20) progressed to stage IV after the initial matching period.

In our final selection of cases control sets from the validation cohort, 154 patients with distant metastasis were included from the 376 eligible cases within our linked source cohort, constituting 41% of the cohort (154/376). Consequently, we sampled 41% of the entire linked source cohort (*n* = 14,198), resulting in a validation cohort of 5,815 stage I/II melanoma patients. This subset is referred to as the nationwide source cohort in Table 1, indicating their random sampling from the national population of stage I/II patients (Table 1).

Within the nationwide source cohort, 49% (*n* = 2,837) of the patients were male and the median age was 62 years (IQR 51–73 years). The median Breslow thickness of the tumors was 0.8 mm (IQR: 0.5–1.4 mm, min–max range: 0.2–14.5) and 9% of the tumors (*n* = 533) was ulcerated. The majority of the tumors were classified as stage I (83%, *n* = 3,804). The patients with stage IV progression (cases) in this cohort were older than all stage I/II patients from the nationwide source cohort (median age 68 years vs. 62 years), had a higher median Breslow thickness (2.7 mm vs. 0.8 mm), and the tumors were more often ulcerated (44% vs. 9%). The metastatic tumors predominantly corresponded to stage II (64%, *n* = 98), while the controls mainly comprised stage I tumors (85%, *n* = 130). The clinical characteristics of the non-matched controls closely mirrored those of the broader nationwide source cohort. In contrary to the discovery set, the differences in age, Breslow thickness, presence of ulceration and AJCC stage between cases and non-matched controls in the validation cohort were statistically significant (Table 1).

The median time to progression to stage IV disease was 1.8 years (IQR: 1.0–3.3, min–max range: 0.3–5.3) among cases, with 98% (*n* = 151) advancing to stage IV within 5 years. At the last follow-up, 29% of the cases (*n* = 44) and 93% of the controls (*n* = 143) were alive. The median follow-up duration until censoring or death was 5.3 years in the source cohort (IQR: 4.0–6.6 years), 3.5 years among the cases (IQR: 1.9–5.4 years) and 6.1 years among the controls (IQR: 4.8–7.3 years). Un update of the data after the initial matching period resulted in at least 2 years follow-up for 98% of the controls (*n* = 151). During this period, 3% (*n* = 4) of the controls progressed to stage III, and one patient progressed to stage IV (Table 1).

### Paired metastatic deposits

Within the discovery set, 59% (*n* = 130) of the 221 cases had at least one histopathologically confirmed distant metastasis in Palga. For these 130 patients, a total of 150 histopathologically confirmed metastatic deposits were identified based on their pathology histories. Thirteen percent (*n* = 17) of these patients had more than one confirmed metastatic site. All corresponding samples were requested through DNTP, and 97 samples were successfully obtained. The most frequently represented sites among these samples were distant lymph nodes, the lungs, and the gastrointestinal tract (Table 2).

**Table 2:** Characteristics of the histopathologically confirmed distant metastases of the cases of the discovery set

	*n* (%)
Number of histopathologically confirmed metastases per patient	
0^1^	71 (32)
1	113 (51)
> 1	17 (8)
Localization histopathologically confirmed distant metastasis^2^	
Lymph node (distant)	15 (15)
Lung	15 (15)
Gastro-intestinal tract	15 (15)
Brains	10 (10)
Bone	6 (6)
Liver	11 (11)
Skin	13 (13)
Other	12 (12)
Total	97 (100)

^1^These patients had non-histologically confirmed metastasis (e.g. confirmed by imaging)

^2^Multiple histopathologically confirmed distant metastasis per patient have been included

## Discussion

The D-ESMEL study addresses the need for enhanced risk stratification in stage I/II melanoma by leveraging a population-based collection of tumor samples, enhanced with clinical, imaging and multi-omics data. Our collection is coupled with an extensive follow-up period and a sufficient number of metastatic events. We assembled a discovery set to identify novel prognostic factors and a validation cohort to build a prognostic model for absolute risk calculations, using nationwide data and primary tumor specimens. Our methodology utilized a case–control approach with population-based data, effectively bridging the gap in the availability of early-stage melanoma specimens from patients who have subsequently developed distant metastases. This provides a solid foundation for developing a prediction model for the absolute risk of distant metastases in stage I/II cutaneous melanoma. Moreover, it serves as a methodological exemplar for effective prognostic cancer research, especially when metastatic events are relatively scarce and advanced molecular techniques are utilized.

### Existing melanoma datasets

The landscape of melanoma research has been enriched by initiatives dedicated to the collection and multi-omics analyses of tumor samples. However, these efforts have been insufficient in predicting the risk of disease progression, particularly in the context of the rare event of metastasis in a large number of thin melanomas. The Cancer Genome Atlas (TCGA) has made significant contributions by generating publicly available genomic, epigenomic, transcriptomic, and proteomic data on melanoma specimens, identifying several genomic and immune subtypes based on these data [24, 25]. However, TCGA predominantly contains metastatic deposits (*n* = 266) rather than primary tumors (*n* = 67), which is an important distinction because genetic mutations may vary between primary tumors and metastatic sites, making metastatic deposits less ideal for studying the prognosis of early-stage melanoma [24, 41]. Additionally, the collection of primary melanomas includes few thin tumors, as the median Breslow thickness is 7.0 mm [24].

The InterMEL collaboration gathers primary melanoma samples (*n* = 685 in their latest publication) based on a case–control design that includes an equal number of cases who died of melanoma within five years and controls who survived disease-free beyond five years [26, 42]. Our data capture longer-term outcomes, with 30% of cases experiencing recurrence after more than five years in the discovery set. Distinctly, InterMEL focuses on stage II-III melanomas, featuring thicker tumors with a median Breslow thickness of 3.8 mm (IQR: 2.5–6.2 mm) [26].

The Lund Melanoma Study group has established BioMEL, a prospective biobank with blood samples and 1 mm punch biopsies of the most aggressive-looking areas suspicious lesions (*n* = 660) [27]. The primary focus of the study is on exploring the molecular differences between nevi and melanomas, as opposed to distinctions between non-metastatic and metastatic melanomas. The prospective nature of the study poses challenges for gathering a sufficient number of metastatic events in early-stage melanomas. Additionally, the biobank relies solely on punch biopsies rather than elliptical excisions. This limits the ability to study the complex interactions within the tumor microenvironment that are better observed in whole-slide images of excisions.

The Leeds Melanoma Cohort is a cohort study that has successfully recruited 2,184 patients with stage I-III primary melanomas between 2000 and 2012 [43–45]. This study is notable for its extensive follow-up period. Based on this dataset, the LMC-150 gene signature was developed [43]. The researchers employed 0.6-mm diameter tissue microarray needles for collecting punch biopsies, which facilitated whole-transcriptome analyses and multiplex immunohistochemistry. The use of small-diameter biopsies restricts the analyses to a limited tissue area and prevents whole-tissue examinations.

### Genetic and immunological risk profiles

Aside from these sample collections, advances have been made to develop and validate prognostic gene expression profiles, and their potential is well-recognized [28–30]. Currently, the most widely published gene expression profiles include the CP-GEP/Merlin™ Assay (SkylineDx B.V.), the 31-GEP Test/DecisionDx-Melanoma (Castle BioSciences Inc.), the 8-GEP Test (MelaGenix) and the Cam_121 (not commercially available). Despite their advancements, these profiles still present limitations in predicting distant metastases with a sufficiently high positive predictive value, particularly for stage I melanoma. The 31-GEP test suffers from a lack of statistical power due to a low number of events in stage I [46–48]. Meanwhile, the 8-GEP test and the Cam_121 were not specifically developed for stage I patients [49, 50]. CP-GEP/Merlin™ Assay was primarily designed for another purpose: to identify patients who may forgo an SLNB and therefore prioritizing a high negative predictive value [51–54].

Melanoma is highly immunogenic and notably responsive to immunotherapy, making its immune landscape promising for revealing additional prognostic information [55]. While the composition of the local immune response has been linked to treatment outcomes, a definitive prognostic immune profile has not been fully established. Within the Leeds Melanoma Cohort, studies have successfully differentiated between groups with poor and good prognoses based on immune cell composition based on transcriptomics, rather than spatial imaging techniques [44, 56] The immunoprint® (Synvie GmbH, Munich, Germany), is a 7-marker singleplex IHC-based signature, and categorizes stage I-III patients into high-risk and low-risk groups. In the most recent validation study, only 37 out of 439 stage I-IIA patients experienced a disease event [57]. Inconsistencies in results remain in other studies analyzing the immune landscape through spatial methods due to a low number of events, the absence of validation studies, and the lack of standardized staining and enumeration methods [58–61].

The genomic landscape of melanoma has also been extensively studied. Whole-genome sequencing has identified significant mutations in genes such as *BRAF*, *CDKN2A*, *NRAS*, and *TP53* [62, 63]. Studies from the Leeds Melanoma Cohort and InterMEL have uncovered additional pathway alterations in primary melanoma [64, 65]. However, the prognostic value of these findings remains uncertain. Notably, genetic variants found in metastatic melanoma samples are often present in their corresponding primary tumors, suggesting that early mutational events may influence metastatic potential [63]. In our study, we focus on these primary tumors. Additionally, we have access to metastatic material for 44% of the cases in our discovery set, which allows us to explore the mutational heterogeneity between primary tumors and their metastases.

Combining data modalities to improve prognostic accuracy is a unique opportunity of the D-ESMEL study. Efforts to create integrated classifiers that combine genomics and imaging data have been reviewed, identifying only nine studies employing multimodal data integration for cancer survival prediction, all lacking external validation [66]. The D-ESMEL study is the first of its kind to integrate whole slide images of H&E, MxIF and IHC, along with RNAseq and DNAseq data, spanning both a discovery set and a validation cohort.

### Strengths and limitations

The strengths of our study include a matched design in the discovery set, the substantial number of metastatic events among stage I melanoma patients due to the use of routinely collected population-based data, a design allowing absolute risk calculations, and the inclusion of patients who progressed after more than five years.

The matched case–control design enables us to identify novel prognostic factors, rather than molecular factors that are strongly related to known prognostic (staging) factors. Matching is a commonly applied strategy to reduce confounding [67]. Although adjustment in multivariable analyses is possible in an unmatched setting, including many variables in a model reduces statistical power.

Using routinely collected healthcare data enables effective capture of metastatic events among early-stage patients. We did not rely on pathology data solely to identify cases, as this can introduce selection bias since not all patients have histopathologically confirmed metastases. Therefore, we also selected patients based on digital patient records. In the validation cohort, this potential bias is addressed as the NCR started using both hospital records and pathology data to record distant metastases since 2017.

For the validation cohort, we employed random sampling from a nationwide population, enabling us to build a risk prediction model for absolute risks – a measure more relevant than relative risks for clinical decision-making [68]. While case–control studies efficiently capture rare events, they are less suitable for validating a risk prediction model if they are not nested within a cohort, because case–control studies are not representative of the general disease population [39]. Therefore, population-based cohorts are considered the optimal study design for developing and validating risk prediction models [39]. However, creating such cohorts can be infeasible and cost-ineffective when generating molecular data from tumor specimens. Instead, a nested case–control design from a well-defined source cohort, with known follow-up data, enables the efficient estimation of odds ratios and calculation of absolute risks [36]. By adjusting the performance metrics properly using the sampling weight of the subjects, we can produce unbiased performance estimates from our validation cohort that are representative of the population cohort. The validation cohort will primarily be used for model development. However, since we included 150% of our targeted sample size, we may use the additional case–control sets for external validation. For instance, the anticipated sample size can be allocated for with 100 case–control sets (derived from approximately 3,800 stage I/II patients) for model development, and the remaining 54 case–control sets (derived from approximately 2,000 stage I/II patients) for external validation. Determining the exact sample size needed for prediction models in advance is challenging, as it depends on the number of predictors, the magnitude of the regression coefficients, and the correlations between predictor variables, which are largely unknown at the study’s inception [69]. Therefore, we may also opt to use the entire validation cohort for model development, in accordance with the original study design.

While other studies included controls with at least five years without metastasis [26], we matched controls on follow-up time to ensure comparability with those included in the risk set of a cohort study. This design acknowledges the possibility that a control may experience distant recurrence after matching. Including controls without metastases for at least five years does not guarantee they will remain metastasis-free, as 30% of our cases in the discovery set developed metastasis after this period. In addition, including controls without metastases for at least five years increases the contrast between cases and controls, which may not reflect a real world cohort. To test our design’s impact, we will update our data annually from the NCR, with the most recent update in February 2024. This allows us to conduct sensitivity analyses limited to case–control sets in which the controls did not develop metastases after the matching period.

A potential limitation of our study is the lack of performed SLNB’s for all patients. The absence of SLNB’s may lead to misclassification of stage III (i.e. locoregional metastatic) disease, as this procedure is used for accurate stage III staging. However, current guidelines do not recommend SLNB for patients with thin (pT1a) melanoma, while patients with stage pT1b or higher are eligible [28]. Before the introduction of systemic therapy for stage III patients in 2019, many eligible patients did not undergo an SLNB, as it had no direct therapeutic implications at the time [4]. Our discovery set, along with the majority of our validation cohort, was selected from a period before 2019. From the stage pT1b patient in the discovery set, 58% did not undergo an SLNB despite being eligible. This proportion remained comparable in the validation cohort (52%). Therefore, limiting our study to those with negative SLNB results would exclude a substantial number of patients, particularly those with thinner pT1a tumors, who constitute a significant portion of the early-stage disease population. Moreover, the SLN positivity rate of patients with pT1b-pT2a tumors is low (~ 10%), minimizing the risk of misclassification in these patients [70]. To ensure the robustness of our findings, we will perform sensitivity analyses on patients who underwent an SLNB and those with a low risk on a positive SLN (pT1a-pT2a). This strategy ensures a comprehensive analysis without excluding a significant segment of the early-stage melanoma population, while also testing the reliability of our results, regardless of whether an SLNB was performed. This is especially relevant for clinical practice, as an SLNB often has no therapeutic consequences and carries the risk of complications [70, 71]. For this reason, advances in gene expression profiling have been made to identify patients who may safely forgo SLNB [72, 73].

We did not use any data on therapy for patient selection, as it could not have influenced the selection process. Systemic therapy is not yet indicated for stage I/II melanoma, and has only been available in the Netherlands for stage III disease since 2019 [4]. Our discovery set was selected from a period before the introduction of adjuvant therapy, meaning patients in this set could have only received systemic therapy after developing distant metastases. The validation cohort, selected between 2016 and 2020, did not include patients with stage III disease at the time of primary diagnosis. We will account for controls who progressed to stage III during follow-up in a sensitivity analysis.

Another potential limitation is the use of aged FFPE material. The use of routinely collected healthcare data allows for the inclusion of patients with long-term progression, but also means that primary tumor material is older (e.g., > 10 years). We could not control the processing and storage conditions of external pathology laboratories, and FFPE material quality can degrade over time, potentially affecting analytical procedures. To avoid antigen degradation, we dipped our IHC/MxIF slides in paraffin. We have implemented quality control measures in our analysis pipelines, and samples failing to meet these criteria will be excluded from further analyses.

## Conclusions

The D-ESMEL study exemplifies effective conduct of prognostic cancer research, particularly when tumor specimens and metastatic events are relatively rare. Our approach demonstrates that molecular prognostic factors can be identified within a matched case–control study and subsequently validated in a nested case–control cohort. This design facilitates the calculation of absolute risks, which is crucial for clinical decision-making. The D-ESMEL study provides a solid foundation for creating a predictive model to determine the absolute risk of distant metastases in stage I/II cutaneous melanoma.

## Supplementary Information

Below is the link to the electronic supplementary material.

Supplementary file1 (DOCX 70 KB)
