# Supplementary material for: The Dutch Early-Stage Melanoma (D-ESMEL) study: a discovery set and validation cohort to predict the absolute risk of distant metastases in stage I/II cutaneous melanoma
Source: Eur J Epidemiol. 2025 Jan 9;40(1):27–42. doi: 10.1007/s10654-024-01188-4 (PMC11799080; doi:10.1007/s10654-024-01188-4)
Supplement: Supplementary file 1 — Supplementary file1 (DOCX 70 KB) [file 10654_2024_1188_MOESM1_ESM.docx]

The Dutch Early-Stage Melanoma Study (D-ESMEL): a Discovery Set and Validation Cohort to Predict the Absolute Risk of Distant Metastases in Stage I/II Cutaneous Melanoma

Catherine Zhou ^1^, Antien L. Mooyaart ^2^, Thamila Kerkour ^1^, Marieke W.J. Louwman ^3^, Marlies Wakkee ^1^, Yunlei Li ^4^, Quirinus J.M. Voorham ^5^, Annette Bruggink ^5^, Tamar E.C. Nijsten ^1^, Loes M. Hollestein ^1,3^*

^1^ Department of Dermatology, Erasmus MC Cancer Institute, Rotterdam, The Netherlands

^2^ Department of Pathology, Erasmus MC Cancer Institute, Rotterdam, The Netherlands

^3^ Department of Research and Development, Netherlands Comprehensive Cancer Organization, Utrecht, The Netherlands

^4^ Department of Pathology & Clinical Bioinformatics, Erasmus MC Cancer Institute, University Medical Center Rotterdam, Rotterdam, The Netherlands

^5^ Dutch Nationwide Pathology Databank (Palga), Houten, The Netherlands

*corresponding author, e-mail address: l.hollestein@erasmusmc.nl, phone: +31 6 50 03 24 07

# Supplementary Information 1


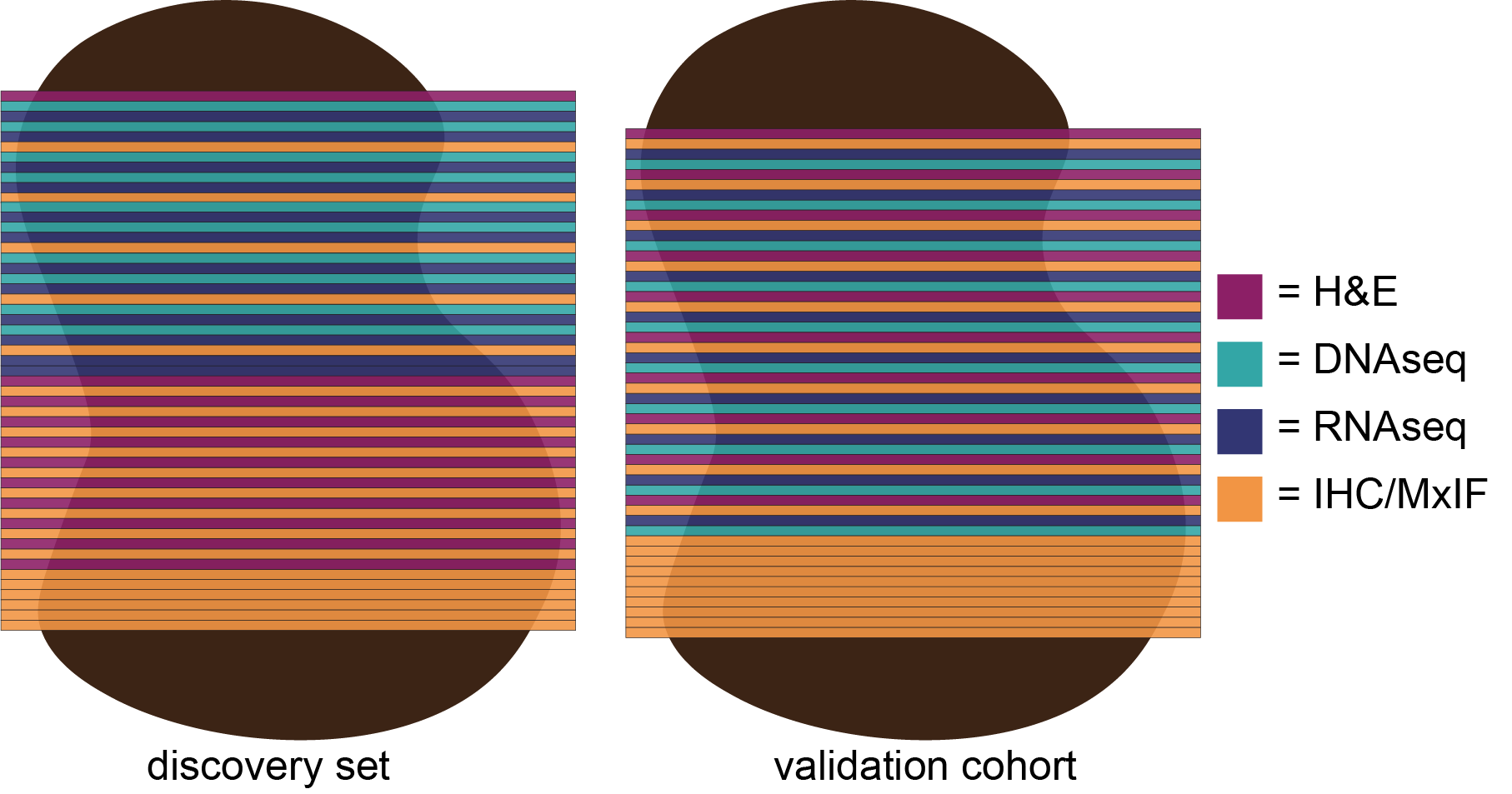


**Supplementary Fig 1.** Sections for different purposes are cut alternately from the tumor, to ensure a more even representation of the tumor in the sections (Abbreviations: H&E = hematoxylin & eosin DNAseq = DNA sequencing, RNAseq = RNA sequencing, IHC = immunohistochemistry, MxIF = multiplex immunofluorescence)
